# Some classes of estimators in the presence of non-response using auxiliary attribute

**DOI:** 10.1186/s40064-016-2922-x

**Published:** 2016-08-05

**Authors:** Saba Riaz, Md. Abud Darda

**Affiliations:** 1 Department of Mathematics and Statistics, Riphah International University, Sector I–14, Islamabad, 44000 Pakistan; 2 Natural Science Academic Group, National University, Gazipur, 1704 Bangladesh

**Keywords:** Proportion, Bias, Mean square error, Ratio function, Exponential function, Efficiency

## Abstract

In this paper, possible solutions of problem of non-response in the variable of interest are proposed when information about an auxiliary attribute is available. By taking motivation from the previous work, modified classes have been suggested for estimating population mean. Two new generalized classes of estimators are presented along with their asymptotic biases and variances. Efficacy analysis of the suggested classes is acquired with the usual regression estimator. Two real examples have been provided to show the efficiency of the proposed design approach and comparison of suggested estimators with the linear regression estimator.

## Introduction and literature review

In sample surveys while estimating an unknown population parameter of the study variable, use of the auxiliary information increases the efficiency of the estimates. In many real scenarios, some auxiliary attributes are highly correlated with the study variable, for instance, person’s weight and gender, cow’s amount of produced milk and breed, crop production and seed type, income and ownership of a house, etc. Many authors have suggested estimators based on auxiliary attributes in simple random sampling or two phase sampling [see Naik and Gupta (1996), Jhajj et al. (2006), Singh et al. (2008), Shabbir and Gupta (2010), Abd-Elfattah et al. (2010), Singh and Solanki (2012), Koyuncu (2012) and references cited therein]. Sometimes people might refuse to reveal their weight or income, or some information are missing because of respondents’ non availability at time of the survey. These situations are related to non-response problem when objects are unavailable or people refuse to answer. In the literature, much work has been done in the existence of non-response problem in the variable of interest and in single or two phase sampling scheme [see Khare and Srivastava (1997), Okafor and Lee (2000), Singh and Kumar (2010), Riaz et al. (2014) and references cited therein].

 Diana et al. (2012) suggested regression type estimators using auxiliary attribute in presence of non-response. According to best of our knowledge little attention is given to the problem of non-response using information on auxiliary attributes.

The aim of this paper is to provide solution to this important problem. At this end, two generalized classes are suggested for the unknown population mean along with the supplementary information about the proportion *P* of the population units having the auxiliary attribute $\phi$. Taking motivation from Shabbir and Gupta (2007), Koyuncu (2012) and Singh and Solanki (2012), some modified classes are also suggested in the existence of missing information.

Let a finite population *U* of *N* distinctive units. Let *Y* be the variable of interest having values $y_i$, $i=(1,\dots ,N)$ with unknown mean $\bar{Y}=\sum _{i=1}^Ny_i/N$ and unknown variance $S_y^2=\sum _{i=1}^N(y_i-\bar{Y})^2/(N-1)$ assuming that non-response occurs in *Y*. Let $\phi$ be the auxiliary attribute correlated with *Y* having values $\phi _i$, $i=(1,\dots ,N)$. Consider $\phi _i=1$, if the* i*th unit of the population has attribute $\phi$ and 0, otherwise. Let $A=\sum _{i=1}^N{\phi _i}$ and $a=\sum _{i=1}^n{\phi _i}$ be the total number of units in the population and in the sample having attribute $\phi$, $P=\left( A/N\right)$ and $\hat{P}=\left( a/n\right)$ denote the proportion of units in the population and in the sample having attribute $\phi$. Let *P* is known and used to estimate the mean $\bar{Y}$. Hansen and Hurwitz (1946) suggested the following sub-sampling technique in presence of non-response. Let a large sample *u* of size *n*$(n<N)$ by simple random sampling without replacement (SRSWOR) to collect information on *Y*. Assuming at first phase, *Y* can be observed only for $n_1$ units out of *n* and, the remaining $n_{2}=n-n_1$ units are taken as non-response. A sub-sample of size $r=n_2/k$, $k>1$ is selected from non-response units where *r* would be an integer or must be rounded. Assuming that all *r* selected units give full response on second call. In this fashion, the population is said to be divided into two groups $U_1$ and $U_2$ of sizes $N_1$ and $N_2$, where $U_1$ is a group of respondents that would give response on the first call at second phase and $U_2$ is non-respondents group which would respond on the second call. Obviously $N_1$ and $N_2$ are unknown quantities.

Considering the above situations, Hansen and Hurwitz (1946) have suggested the following estimator for population mean,

1 $$\begin{aligned} \bar{y}^*=d_1\bar{y}_1+d_2\bar{y}_{2r}, \end{aligned}$$

where

$$\begin{aligned} \bar{y}_1=\dfrac{\sum ^{n_1}_{i=1}y_i}{n_1}, \quad \bar{y}_{2r}=\dfrac{\sum ^{r}_{i=1}y_i}{r}, \quad d_1=\dfrac{n_1}{n} \quad \text {and} \quad d_2=\dfrac{n_2}{n}. \end{aligned}$$

As well known $\bar{y}^*$ is an unbiased estimator of $\bar{Y}$

$$\begin{aligned} E(\bar{y}^*)=D_1\bar{Y}_1+D_2\bar{Y}_2=\bar{Y}, \end{aligned}$$

where

$$\begin{aligned} \bar{Y}_1=\dfrac{\sum ^{N_1}_{i=1}y_i}{N_1} ,\quad \bar{Y}_2=\dfrac{\sum ^{N_2}_{i=1}y_i}{N_2} ,\quad D_1=\dfrac{N_1}{N} \quad \text {and} \quad D_2=\dfrac{N_2}{N}. \end{aligned}$$

The variance of $\bar{y}^*$ is given by

2 $$\begin{aligned} \text {Var}(\bar{y}^*)=[{\theta }S_y^2+{\lambda }S_{y(2)}^2]=\widetilde{S}_y^2, \end{aligned}$$

where

$$\begin{aligned} S_{y(2)}^2=\dfrac{\sum _{i=1}^{N_2}(y_i-\bar{Y}_2)^2}{N_2-1}, \quad \theta =\left( \dfrac{1}{n}-\dfrac{1}{N}\right) \quad \text {and} \quad \lambda =\dfrac{N_2(k-1)}{nN}. \end{aligned}$$

We can write (2) as

$$\begin{aligned} \text {Var}(\bar{y}^*)=\widetilde{S}_y^2=\bar{Y}^2\widetilde{C}_y^2. \end{aligned}$$

If *P* is known, we can first of all consider the regression estimator of $\bar{Y}$

3 $$\begin{aligned} \bar{y}^*_{\text {reg}}=\bar{y}^*+w(P-\hat{P}), \end{aligned}$$

where *w* is an unknown constant to be selected properly.

The mean square error (MSE) of $\bar{y}^*_{\text {reg}}$ is minimum when

$$\begin{aligned} w=\dfrac{S_{y{\phi }}}{S^2_{\phi }}=w^{o} \text {(say)}. \end{aligned}$$

The minimum MSE of $\bar{y}^*_{\text {reg}}$ is given by

4 $$\begin{aligned} \text {minMSE}\left( \bar{y}^*_{\text {reg}}\right) ={\theta }{S}_y^2\left( 1-{\rho }^2_{pb}\right) +{\lambda }S^2_{y(2)}, \end{aligned}$$

where

$$\begin{aligned} {\rho ^2_{pb}}=\dfrac{S^2_{y{\phi }}}{S^2_yS^2_{\phi }}, \quad S_{\phi }^2=\dfrac{\sum _{i=1}^N({\phi _i}-P)^2}{N-1} \quad \text {and} \quad S_{y{\phi }}=\dfrac{\sum _{i=1}^N(y_i-\bar{Y})({\phi _i}-P)}{N-1}. \end{aligned}$$

We can write (4) as

5 $$\begin{aligned} \text {minMSE}\left( \bar{y}^*_{\text {reg}}\right) ={\theta }{\bar{Y}^2}{C}_y^2\left( 1-{\rho }^2_{pb}\right) +{\lambda }{\bar{Y}^2}C^2_{y(2)}, \end{aligned}$$

where

$$\begin{aligned} C^2_y=\dfrac{S^2_y}{\bar{Y}^2}, \quad C^2_{y(2)}=\dfrac{S^2_{y(2)}}{\bar{Y}^2}, \quad {\rho ^2_{pb}}=\dfrac{C^2_{yp}}{C^2_yC^2_p}, \quad C_p^2=\dfrac{S_{\phi }^2}{P^2} \quad \text {and} \quad C_{yp}=\dfrac{S_{y{\phi }}}{\bar{Y}P}. \end{aligned}$$

## Modified classes

In this section, we have considered Shabbir and Gupta (2007), Koyuncu (2012) and Singh and Solanki (2012) classes because those are more efficient than the regression estimator. Shabbir and Gupta (2007) suggested a class of ratio estimator for the population mean $\bar{Y}$ using known information of the auxiliary attribute. Later Singh and Solanki (2012) and Koyuncu (2012) proposed classes on the same subject using different known population parameters such as $(\rho _{pb}, \; C_p)$ etc. Therefore, taking motivation from the work of the just quoted authors, we modify their classes in frame work of non-response on *Y* .

### First modified class

Shabbir and Gupta (2007) proposed the class of estimators

6 $$\begin{aligned} \bar{y}_{\text {SG}}=\bar{y}\left[ w_1+w_2(P-\hat{P})\right] \left( \dfrac{P}{\hat{P}}\right) , \end{aligned}$$

where $w_1$ and $w_2$ are suitable weights.

If we assume that there is non-response on Y, the class (6) can be modified as

7 $$\begin{aligned} \bar{y}^*_{\text {M1}}=\bar{y}^*\left[ w_1+w_2(P-\hat{P})\right] \left( \dfrac{P}{\hat{P}}\right) . \end{aligned}$$

For an easy computation of the bias (B) and the mean square error (MSE) of $\bar{y}^*_{\text {M1}}$ up to first order approximation, it is convenient to express (7) in terms of $\delta$’s

8 $$\begin{aligned} \bar{y}^*_{\text {M1}}=\bar{Y}\left( 1+{\delta }^*_y\right) \left[ w_1-w_2P{\delta }_{\phi }\right] \left( 1+{\delta }_{\phi }\right) ^{-1}, \end{aligned}$$

where

$$\begin{aligned} \delta _y^*&= {} \dfrac{\bar{y}^*-\bar{Y}}{\bar{Y}},\\ \delta _{\phi }&= {} \dfrac{\hat{P}-P}{P} \end{aligned}$$

and then use Taylor expansion of (8). So, remembering that

$$\begin{aligned} E(\delta _y^*)&= {} E(\delta _{\phi })=0,\\ E(\delta _y^{*2})&= {} \mathbf {C}_y^2, \quad E(\delta _{\phi }^2)={\theta }C_{p}^2, \quad E({\delta _y^*}{\delta _{\phi }})={\theta }C_{yp}, \end{aligned}$$

we have

9 $$\begin{aligned} \text {B}(\bar{y}^*_{\text {M1}})=\bar{Y}\left[ \left( w_1-1\right) +{\theta } \left( w_1+w_2P\right) \left( C^2_p-C_{yp}\right) \right] \end{aligned}$$

and

10 $$\begin{aligned} \text {MSE}(\bar{y}^*_{\text {M1}})=\bar{Y}^2\left[ \left( w_1-1\right) ^2+w_1^2 \widetilde{C}_y^2+{\theta }\left( w_1+w_2P\right) \left\{ C_p^2\left( 3w_1+w_2P-2\right) +2C_{yp}\left( 1-2w_1\right) \right\} \right] . \end{aligned}$$

The mean square error of $\bar{y}^*_{\text {M1}}$ will be minimum

$$\begin{aligned} w_1=\dfrac{{\theta }C_p^4-C_p^2\left( 1+3{\theta }C_{yp}\right) +2{\theta }C^2_{yp}}{\left[ {\theta }C_p^4-C_p^2(1+\mathbf {C}_y^2+4{\theta }C_{yp})+4{\theta }C_{yp}^2\right] } =w_1^{o} \text {(say)} \end{aligned}$$

and

11 $$\begin{aligned} w_2&= {} \dfrac{\left( C_{yp}-C^2_p\right) \left( {\theta }C_p^2+C^2_y-2{\theta } C_{yp}-1\right) }{P\left[ {\theta }C_p^4-C_p^2(1+\mathbf {C}_y^2+4{\theta }C_{yp}) +4{\theta }C_{yp}^2\right] }=w_2^{o} \text {(say)},\nonumber \\ \text {minMSE}(\bar{y}^*_{\text {M1}})&= {} \dfrac{\bar{Y}^2\left[ {\theta }\mathbf {C}_y^2C_p^4 -\mathbf {C}_y^2C_p^2(1+2{\theta }C_{yp})+{\theta }C_{yp}^2(1+\mathbf {C}_y^2)\right] }{\left[ {\theta } C_p^4-C_p^2(1+\mathbf {C}_y^2+4{\theta }C_{yp})+4{\theta }C_{yp}^2\right] }. \end{aligned}$$

### Second modified class

Koyuncu (2012) suggested a regression-cum-ratio class

12 $$\begin{aligned} \bar{y}_\text {K}=\left[ w_1\bar{y}+w_2(P-\hat{P})\right] \left( \dfrac{{\eta }P+\psi }{\eta {\hat{P}}+\psi }\right) , \end{aligned}$$

where $w_1$ and $w_2$ having the same expressions defined earlier, $\eta$ and $\psi$ are either real numbers or functions of the known parameter associated with an auxiliary attribute such as $C_p$, $\beta _2(\phi )$ and $\rho _{pb}$. Of course the aim was to increase the performance using more information.

We can modify the class (12), assuming non-response on *Y*

13 $$\begin{aligned} \bar{y}^*_\text {M2}=\left[ w_1\bar{y}^*+w_2(P-\hat{P})\right] \left( \dfrac{{\eta }P +\psi }{\eta {\hat{P}}+\psi }\right) . \end{aligned}$$

The bias and the MSE of $\bar{y}^*_\text {M2}$ to the first order of approximation can be written as

14 $$\begin{aligned} \text {B}(\bar{y}^*_{\text {M2}})=\bar{Y}\left[ \left( w_1-1\right) +{\theta } \left( w_1{\tau }^2+w_2{\tau }P\right) C^2_p-{\theta }w_1{\tau }C_{yp}\right] \end{aligned}$$

and

15 $$\begin{aligned} \begin{aligned} \text {MSE}(\bar{y}^*_{\text {M2}})&=\bar{Y}^2\left( w_1-1\right) ^2+w_1^2\bar{Y}^2\mathbf {C}^2_y +-2{\theta }w_1\bar{Y}C_{yp}\left[ w_2P+\bar{Y}P{\tau }(2w_1-1)\right] \\&+{\theta }C^2_p\left[ w_1\bar{Y}^2{\tau }^2\left( 3w_1-2\right) +w_2^2P^2+2(2w_1-1) w_2\bar{Y}P{\tau }\right] , \end{aligned} \end{aligned}$$

where $$\tau =\dfrac{{\eta }P}{{\eta }P+\psi }$$.

The mean square error of $\bar{y}^*_{\text {M2}}$ is minimized for

$$\begin{aligned} w_1=\dfrac{C^2_p\left( {\theta }{\tau }^2C^2_p-1\right) }{{\theta }{\tau }^2C^4_p-C^2_p (1+\mathbf {C}^2_y)+{\theta }C^2_{yp}}=w_1^{o} \text {(say)} \end{aligned}$$

and

$$\begin{aligned} w_2=\dfrac{\bar{Y}\left[ {\tau }C^2_p(1+{\theta }{\tau }C_{yp}-\mathbf {C}^2_y)-{\theta }{\tau }^3 C^4_p+C_{yp}({\theta }{\tau }C_{yp}-1)\right] }{P\left[ {\theta }{\tau }^2 C^4_p-C^2_p(1+\mathbf {C}^2_y)+{\theta }C^2_{yp}\right] }=w_2^{o} \text {(say)}. \end{aligned}$$

To emphasize the comparison with the regression estimator (3), we can express the minimum MSE of $\bar{y}^*_{\text {M2}}$ as

16 $$\begin{aligned} \text {minMSE}(\bar{y}^*_\text {M2}) = \dfrac{\left( 1-{\theta }{\tau ^2}C_p^2\right) \text {MSE}(\bar{y}^*_\text {reg})}{\left( 1-{\theta }{\tau ^2}C_p^2\right) +\dfrac{{\text {MSE}(\bar{y}^*_\text {reg})}}{{\bar{Y}^2}}}. \end{aligned}$$

### Third modified class

Singh and Solanki (2012) proposed the class

17 $$\begin{aligned} \bar{y}_{\text {SS}}=\bar{y}\left[ w_1+w_2(P-\hat{P})\right] \left( \dfrac{{\eta }P +{\delta }{\psi }}{{\eta }{\hat{P}}+{\delta }{\psi }}\right) ^{\gamma }, \end{aligned}$$

where $\gamma$ is a real number, $\delta$ is an integer which takes values +1 and -1 for designing the estimators and keeping $(w_1 , \; w_2, \; \eta , \; \psi )$ same as defined before. Note that Shabbir and Gupta (2007) class is a member of this class.

We can modify the class (17) considering incomplete information on *Y*

18 $$\begin{aligned} \bar{y}^*_{\text {M3}}=\bar{y}^*\left[ w_1+w_2(P-\hat{P})\right] \left( \dfrac{{\eta }P+{\delta }{\psi }}{{\eta }{\hat{P}}+{\delta }{\psi }}\right) ^{\gamma }. \end{aligned}$$

The bias and MSE of $\bar{y}^*_{\text {M3}}$ to the first order of approximation are given by

19 $$\begin{aligned} \text {B}(\bar{y}^*_{\text {M3}})=\bar{Y}\left[ (w_1-1)+{\theta }C_p^2\left\{ {\gamma }{\nu } \left( w_2P+\dfrac{w_1(\gamma +1)}{2}\right) -\left( w_2P+w_1\gamma {\nu }\right) k_p\right\} \right] \end{aligned}$$

and

20 $$\begin{aligned} \text {MSE}(\bar{y}^*_{\text {M3}})=\bar{Y}^2\left[ 1+w_1^2A^*+w_2^2B^*+2w_1w_2C^*-2w_1 D^*-2w_2E^*\right] , \end{aligned}$$

where

$$\begin{aligned} k_p&= {} {\rho _{pb}}\dfrac{\mathbf {C}_y}{C_p}, \quad \nu = \dfrac{{\eta }P}{{\eta }P+{\delta }{\psi }},\\ A^*&= {} 1+\mathbf {C}_y^2+{\theta }{\gamma }{\nu }C_p^2\left[ (2\gamma +1)\nu -4k_p\right] ,\\ B^*&= {} {\theta }P^2C_p^2 , \quad C^*=2{\theta }PC_p^2\left( {\gamma }{\nu }-k_p\right) ,\\ D^*&= {} 1+{\theta }{\gamma }{\nu }C_p^2\left( \dfrac{({\gamma }+1)\nu }{2}-k_p\right) , \quad E^*={\theta }PC_p^2\left( {\gamma }{\nu }-k_p\right) . \end{aligned}$$

Minimizing the $\text {MSE}(\bar{y}^*_{\text {M3}})$ to achieve optimum values of constants $w_1$ and $w_2$

$$\begin{aligned} w_1=\dfrac{B^*D^*-C^*E^*}{A^*B^*-C^{*2}}=w_1^{o} \text {(say)} \end{aligned}$$

and

21 $$\begin{aligned} w_2&= {} \dfrac{A^*E^*-C^*D^*}{A^*B^*-C^{*2}}=w_2^{o} \text {(say)},\nonumber \\ \text {minMSE}(\bar{y}^*_{\text {M3}})&= {} \bar{Y}^2\left( 1-\dfrac{B^*D^{*2}-2C^*D^*E^*+A^*E^{*2}}{A^*B^*-C^{*2}}\right) . \end{aligned}$$

## Suggested classes

In this section, we have introduced two general classes of estimators for the population mean assuming (as in the previous Section) non-response occurs in the study variable with known information on the auxiliary attribute. The first suggested class is obtained starting from a generalization of the third modified class, defined in (18), while the second class is the result of motivation from Diana et al. (2011).

### First class

Let

22 $$\begin{aligned} \bar{y}_{\text {S1}}=\bar{y}^*(w_1+w_2u)g(u), \end{aligned}$$

where $u=(P-\hat{P})$, $w_1$ and $w_2$ are constants to be chosen properly and *g* is a generic function that satisfy the following mild conditions

- *g* is continuous and bounded in a neighborhood of zeros.
- *g* does not depend on *n*, *N* and $(\phi _1,\dots ,\phi _N)$.
- *g* is a three times differentiable function with continuous and bounded derivatives.

Expanding *g*(*u*) using Taylor’s series up to order $o_p(u^2)$, the resulting expression for the class is given by

23 $$\begin{aligned} \bar{y}_{\text {S1}} \cong \bar{y}^*\left( w_1+w_2u\right) \left[ g(0)+g'(0)u+\dfrac{1}{2}g''(0)u^2\right] , \end{aligned}$$

where *g*(0) is a constant term, $g'(0)$ is first order partial derivative of *g*(*u*) in zero and $g''(0)$ is second order partial derivative in zero. For sake of simplicity, we can write $g(0)=a_0$, $g'(0)=b_0$ and $\dfrac{1}{2}g''(0)=c_0$.

Now expressing (23) in terms of $\delta$’s

24 $$\begin{aligned} \bar{y}_{\text {S1}} \cong \bar{Y}\left( 1+\delta ^*_y\right) \left( w_1-w_2P{\delta _{\phi }}\right) \left( a_0-b_0P{\delta _{\phi }} +c_0P^2{\delta ^2_{\phi }}\right) . \end{aligned}$$

The bias and the MSE of $\bar{y}_{\text {S1}}$ up to the first order of approximation are

25 $$\begin{aligned} \text {B}(\bar{y}_{\text {S1}}) = \bar{Y}\left[ (w_1a_0-1)+{\theta }\left\{ (w_1c_0+w_2b_0)P^2C_p^2-(w_1b_0+w_2a_0) PC_{yp}\right\} \right] \end{aligned}$$

and

26 $$\begin{aligned} \begin{aligned} \text {MSE}(\bar{y}_{\text {S1}})&=\bar{Y}^2\left[ (w_1a_0-1)^2+w_1^2a_0^2\mathbf {C}_y^2 +{\theta }\left\{ w_1^2\left( b_0^2P^2C_p^2-2a_0b_0PC_{yp}\right) \right. \right. \\&\quad \left. \left. +(2w_1b_0+w_2a_0)w_2a_0P^2C_p^2 -2w_2(w_1a_0-1)\left( a_0PC_{yp}-b_0P^2C_p^2\right) \right. \right. \\&\quad \left. \left. +2w_1(w_1a_0-1)\left( c_0P^2C_p^2-b_0PC_{yp}\right) \right\} \right] . \end{aligned} \end{aligned}$$

Minimizing (26) with respect to $w_1$ and $w_2$, we obtain

$$\begin{aligned} w_1=\dfrac{U^*_1+U^*_2}{a_0\left( T^*_1+T^*_2\right) }=w_1^{o} \text {(say)} \end{aligned}$$

and

$$\begin{aligned} w_2=\dfrac{\bar{Y}V^*_1+V^*_2}{a_0^2P\left( T^*_1+T^*_2\right) }=w_2^{o} \text {(say)}, \end{aligned}$$

where

$$\begin{aligned} T^*_1&= a_0^2\left[ \bar{Y}^2C_p^2+\bar{Y}^2\left( \mathbf {C}_y^2C_p^2-{\theta }C_{yp}^2\right) \right] +{\theta }\bar{Y}^2P^2C_p^4\left( 2a_0c_0-3b_0^2\right) ,\\ U^*_1&= C_p^2\left[ a_0^2+{\theta }\left( a_0c_0P^2C_p^2-2b_0^2P^2C_p^2\right) \right] ,\\ V^*_1&= a_0^3C_{yp}+a_0^2P\left[ b_0\left( \mathbf {C}_y^2C_p^2-{\theta }C_{yp}^2-C_p^2\right) +{\theta }c_0PC_p^2C_{yp}\right] \\& \quad +{\theta }b_0^3P^3C_p^4-2{\theta }a_0b_0^2P^2C_p^2C_{yp},\\ T^*_2&= 4{\theta }a_0b_0\bar{Y}^2PC_p^2C_{yp}-3{\theta }a_0^2\bar{Y}^2C_{yp}^2,\\ U^*_2&= 3{\theta }a_0b_0PC_p^2C_{yp}-2{\theta }a_0^2C_{yp}^2 \end{aligned}$$

and

$$\begin{aligned} V^*_2=3{\theta }a_0^2b_0PC_{yp}^2-{\theta }a_0^3C^2_yC_{yp}+{\theta }a_0b_0^2 P^2C_p^2C_{yp}. \end{aligned}$$

Hence, with the help of above notations, one can write minimum MSE of $\bar{y}_\text {S1}$ as follows

27 $$\begin{aligned} \text {minMSE}(\bar{y}_{\text {S1}}) = \dfrac{\bar{Y}^2\left( W^*_1+W^*_2\right) }{a_0^2\left( T^*_1+T^*_2\right) }, \end{aligned}$$

where

$$\begin{aligned} \begin{aligned} W^*_1&=\bar{Y}^2\left[ a_0^4\left( \mathbf {C}_y^2C_p^2-{\theta }C_{yp}^2\right) +{\theta }^2b_0^2 P^4C_p^6\left( 2a_0c_0-b_0^2\right) \right. \\&\left. -{\theta }a_0^2P^2C_p^2\left\{ b_0^2\left( \mathbf {C}_y^2C_p^2-{\theta }C_{yp}^2\right) +{\theta }c_0^2P^2C_p^4\right\} \right] \end{aligned} \end{aligned}$$

and

$$\begin{aligned} \begin{aligned} W^*_2&=\bar{Y}^2\left[ 2{\theta }^2a_0b_0^3P^3C_p^4C_{yp}-2{\theta }^2a_0^2b_0c_0P^3C_p^4C_{yp}-{\theta }a_0^4\mathbf {C}_y^2C_{yp}^2 \right. \\\quad &\left. -3{\theta }^2a_0^2b_0^2P^2C_p^2C_{yp}^2+2{\theta }a_0^3b_0P\mathbf {C}_y^2C_p^2C_{yp}+2{\theta }^2a_0^3c_0P^2C_p^2C_{yp}^2\right] . \end{aligned} \end{aligned}$$

### Second class

Motivated by Diana et al. (2011), we consider the class

28 $$\begin{aligned} \bar{y}_\text {S2}=\left( w_1\bar{y}^*+w_2u\right) g(u), \end{aligned}$$

where *u* and *g*(*u*) are explained in earlier section.

If we expand *g*(*u*) again using Taylor’s series, $\bar{y}_{\text {S2}}$ becomes

29 $$\begin{aligned} \bar{y}_{\text {S2}} \cong \left( \bar{y}^*w_1+w_2u\right) \left[ g(0)+g'(0)u+\dfrac{1}{2}g''(0)u^2\right] . \end{aligned}$$

The bias and the MSE to the first order of approximation can be written as

30 $$\begin{aligned} \text {B}(\bar{y}_\text {S2}) = \bar{Y}\left( a_0w_1-1\right) +{\theta }\left[ (w_1\bar{Y}c_0+w_2b_0)P^2C_p^2-w_1 \bar{Y}b_0PC_{yp}\right] \end{aligned}$$

and

31 $$\begin{aligned} \begin{aligned} \text {MSE}(\bar{y}_\text {S2})&=\bar{Y}^2(a_0w_1-1)^2+\left[ w_1^2a_0^2\bar{Y}^2\mathbf {C}_y^2 +{\theta }P^2C_p^2 \left\{ w_2^2a_0^2 \right. \right. \\&\quad \left. \left. +2w_1a_0\bar{Y}(2w_2b_0+w_1c_0\bar{Y}) +\bar{Y}\left( w_1^2b_0^2\bar{Y}-2w_2b_0-2w_1c_0\bar{Y}\right) \right\} \right. \\&\quad \left. -2{\theta }w_1\bar{Y}PC_{yp}\left( w_2a_0^2+2w_1a_0b_0\bar{Y}-b_0\bar{Y}\right) \right] . \end{aligned} \end{aligned}$$

By minimizing $\text {MSE}(\bar{y}_\text {S2})$, one can get the optimum values of the constants $w_1$ and $w_2$

$$\begin{aligned} w_1=\dfrac{U_1^*}{a_0T_1^*}=w_1^{o} \text {(say)} \end{aligned}$$

and

$$\begin{aligned} w_2=\dfrac{\bar{Y}V_1^*}{a_0^2PT^*_1}=w_2^{o} \text {(say)}, \end{aligned}$$

Therefore,

32 $$\begin{aligned} \text {minMSE}(\bar{y}_\text {S2})=\dfrac{\bar{Y}^2W^*_1}{a_o^2T^*_1}. \end{aligned}$$

It is observed that Rao (1991) class is a member of Diana et al. (2011). Also in our case, if considered *g*(*u*) appears as an identity function, the class (28) reduces to

33 $$\begin{aligned} \bar{y}^*_{\text {S2(R)}}=w_1\bar{y}^*+w_2u, \end{aligned}$$

that is the corresponding version of Rao (1991) class when non-response is present.

The bias and the MSE of $\bar{y}^*_{\text {S2(R)}}$ can be written as

34 $$\begin{aligned} \text {B}\left( \bar{y}^*_{\text {S2(R)}}\right) =\bar{Y}(w_1-1) \end{aligned}$$

and

35 $$\begin{aligned} \text {MSE}\left( \bar{y}^*_{\text {S2(R)}}\right) = \bar{Y}^2(w_1-1)^2+w_1^2\bar{Y}^2\mathbf {C}_y^2+{\theta }\left( w_2^2P^2C_p^2-2w_1w_2 \bar{Y}PC_{yp}\right) . \end{aligned}$$

The optimum values of constants $w_1$ and $w_2$ are

$$\begin{aligned} w_1=\dfrac{C_p^2}{C_p^2+\left( \mathbf {C}_y^2C_p^2-{\theta }C_{yp}^2\right) }=w_1^{o} \text {(say)} \end{aligned}$$

and

$$\begin{aligned} w_2=\dfrac{\bar{Y}C_{yp}}{P\left[ C_p^2+\left( \mathbf {C}_y^2C_p^2-{\theta }C_{yp}^2\right) \right] }=w_2^{o} \text {(say)}. \end{aligned}$$

The minimum MSE of $\bar{y}^*_{\text {S2(R)}}$ is given by

36 $$\begin{aligned} \text {minMSE}\left( \bar{y}^*_{\text {S2(R)}}\right) =\dfrac{\text {MSE}\left( \bar{y}^*_{\text {reg}}\right) }{1+\dfrac{\text {MSE}\left( \bar{y}^*_{\text {reg}}\right) }{\bar{Y}^2}}. \end{aligned}$$

From (36) is clear that the estimator $\bar{y}^*_{\text {S2(R)}}$ performs always better than $\bar{y}^*_{\text {reg}}$, in their optimal case.

From (16) and (36), it is easy to see that the minimum MSE of $\bar{y}^*_{\text {M2}}$ becomes equal to the minimum MSE of $\bar{y}^*_{\text {S2(R)}}$ when $\tau =0$. One can also observe that regression-cum-ratio estimator (13) may perform better than regression estimator for different choices of $\tau$. Note that the MSE of $\bar{y}_{\text {S2}}$ looks like the MSE of Diana et al. (2011), however, their work is related to complete information for the study variable *Y* and the auxiliary variable *X*. But the class $\bar{y}_{\text {S2}}$ highlights the non-response problem especially when someone estimates the population mean using information of the auxiliary attribute.

## Choice of function *g*

The performance of the proposed classes $\bar{y}_{\text {S1}}$ and $\bar{y}_{\text {S2}}$ depends upon the selection of function *g*. Careful choice for *g* is a crucial factor and it requires deep insight both from theoretical and practical point of view. There are many possible choices for *g* but we consider only ratio and exponential function, because they are found good choices from both theoretical and practical point of view.

(i): Consider a ratio type function suggested by Singh and Solanki (2012) assuming $\gamma =1$
  37 $$\begin{aligned} g(u)=\left( \dfrac{{\eta }P+{\delta }{\psi }}{{\eta }P+{\delta }{\psi }-{\eta }u}\right) . \end{aligned}$$

Expanding *g*(*u*) by Taylor’s theorem, we get

$$\begin{aligned} a_0=g(0)=1, \quad \quad b_0=g'(0)=\dfrac{\eta }{{\eta }P+{\delta }{\psi }}, \quad \quad c_0=\dfrac{1}{2}g''(0)=\dfrac{{\eta }^2}{({\eta }P+{\delta }{\psi })^2}. \end{aligned}$$

When $\delta =1$, *g*(*u*) is similar to function considered by Koyuncu (2012).

When $\eta =1$ and $\psi =0$ then *g*(*u*) become similar to the ratio function suggested by Shabbir and Gupta (2007).

The suggested classes $\bar{y}_{\text {S1}}$ and $\bar{y}_{\text {S2}}$ become

$$\begin{aligned} \bar{y}^*_{\text {S1(1)}}=\bar{y}^*(w_1+w_2u)\left( \dfrac{{\eta }P+{\delta }{\psi }}{{\eta }{{P}}+{\delta }{\psi }-{\eta }u}\right) \end{aligned}$$

and

$$\begin{aligned} \bar{y}^*_{\text {S2(1)}}=(w_1\bar{y}^*+w_2u)\left( \dfrac{{\eta }P+{\delta } {\psi }}{{\eta }{{P}}+{\delta }{\psi }-{\eta }u}\right) . \end{aligned}$$

If we consider $\eta =1$ and $\psi =0$, $\bar{y}^*_{\text {S1(1)}}$ is equivalent to $\bar{y}^*_{\text {M1}}$ and $\bar{y}^*_{\text {S2(1)}}$ is equal to $\bar{y}^*_{\text {M2}}$, for $\delta =1$.

Hence, we can conclude that $(\bar{y}^*_{\text {M1}}, \; \bar{y}^*_{\text {M3}})$ belong to the class $\bar{y}_{\text {S1}}$ and $\bar{y}^*_{\text {M2}}$ is a member of the class $\bar{y}_{\text {S2}}$.

(ii): Consider an exponential function
  38 $$\begin{aligned} g(u)=\exp \left( \dfrac{u}{2P-u}\right) . \end{aligned}$$

Using Taylor’s theorem to expand *g*(*u*), we have

$$\begin{aligned} a_0=1, \quad b_0=\dfrac{1}{2P},\quad c_0=\dfrac{3}{8P^2}. \end{aligned}$$

Then $\bar{y}_{\text {S1}}$ and $\bar{y}_{\text {S2}}$ can be written as

$$\begin{aligned} \bar{y}^{*}_{\text {S1(2)}}=\bar{y}^*(w_1+w_2u)\exp \left( \dfrac{u}{2P-u}\right) \end{aligned}$$

and

$$\begin{aligned} \bar{y}^{*}_{\text {S2(2)}}=(w_1\bar{y}^*+w_2u)\exp \left( \dfrac{u}{2P-u}\right) . \end{aligned}$$

The minimum MSE of $\bar{y}^{*}_{\text {S1(2)}}$ and $\bar{y}^{*}_{\text {S2(2)}}$ are given by

$$\begin{aligned} \text {minMSE}\left( \bar{y}^{*}_{\text {S1(2)}}\right) =\dfrac{\bar{Y}^2\left[ {\theta }^2C_p^6+8{\theta } C_p^4\left( {\theta }C_{yp}+2\mathbf {C}_y^2\right) -16C_p^2\left\{ 4\mathbf {C}_y^2(1+{\theta }C_{yp})+{\theta }^2 C_{yp}^2\right\} +64{\theta }C_{yp}^2\left( 1+\mathbf {C}_y^2\right) \right] }{64\left[ 4{\theta }C_{yp}^2-C_p^2 \left( 1+\mathbf {C}_y^2+2{\theta }C_{yp}\right) \right] } \end{aligned}$$

and

$$\begin{aligned} \text {minMSE}\left( \bar{y}^{*}_{\text {S2(2)}}\right) =\dfrac{\bar{Y}^2\left[ \left( \mathbf {C}_y^2C_p^2 -{\theta }C_{yp}^2\right) \left( 64{\theta }-16{\theta }C_p^2\right) -{\theta }C_p^6\right] }{64\left[ C_p^2+\mathbf {C}_y^2C_p^2 -{\theta }C_{yp}^2\right] }. \end{aligned}$$

## Efficiency comparisons

In this section, efficiency of the proposed estimators on the basis of their minimum mean square error has been evaluated by analyzing the performance of estimators, when possible, specially numerically. It is well known that the regression estimator $\bar{y}^*_{\text {reg}}$ is always more efficient than the Hansen and Hurwitz (1946) estimator (for instance, see (2) and (4)). For this reason we make efficiency comparison of the proposed classes with the regression estimator.

From the comparison of (4) with (32), after some computation, one can get

$$\begin{aligned} \text {MSE}\left( \bar{y}^*_{\text {reg}}\right) -\text {minMSE}(\bar{y}_{\text {S2}})\ge 0, \end{aligned}$$

when

$$\begin{aligned} \dfrac{\left[ a_0^4\text {MSE}\left( \bar{y}^*_{\text {reg}}\right) -{\theta }\bar{Y}^2P^2C_p^2 \left( b_0^2-a_0c_0\right) \right] ^2}{a^2_0\left[ a_0^2\left\{ \bar{Y}^2+\text {MSE}\left( \bar{y}^*_{\text {reg}}\right) \right\} +{\theta }\bar{Y}^2P^2C_p^2\left( 2a_0c_0-3b_0^2\right) \right] } \ge 0. \end{aligned}$$

This expression will be certainly $> 0$ if $\left( 2a_0c_0-3b_0^2\right) \ge 0$ and hence $\bar{y}_{\text {S2}}$ is more efficient than the regression estimator.

Now making comparison of $\bar{y}^*_{\text {M2}}$ and $\bar{y}^*_{\text {S2}(R)}$ with $\bar{y}^*_{\text {reg}}$

$$\begin{aligned} \text {MSE}\left( \bar{y}^*_{\text {reg}}\right) -\text {minMSE}\left( \bar{y}^*_{\text {M2}}\right) \ge 0 \end{aligned}$$

and

$$\begin{aligned} \text {MSE}\left( \bar{y}^*_{\text {reg}}\right) -\text {minMSE}\left( \bar{y}^*_{\text {S2}(R)}\right) \ge 0. \end{aligned}$$

### *Remark*

It can be observed from (4) and (16) and, from (4) and (36) respectively, that these expressions are always positive. Furthermore, it is not easy to make analytical comparison for $\left( \bar{y}^*_{\text {M1}}, \; \bar{y}^*_{\text {M3}}, \; \bar{y}_{\text {S1}}\right)$.

So in this Section, we make numerical comparison of modified and suggested classes using two population data sets as earlier considered by Shabbir and Gupta (2007), Abd-Elfattah et al. (2010) and Koyuncu (2012).

### Population I

[Source: Sukhatme and Sukhatme (1970), p. 256]

$$\begin{aligned} y&= {} \text {Number of villages in the circles}.\\ \phi&= {} \text {A circle consisting of more than five villages}.\\ N&= {} 89, \quad \quad n=23, \quad \quad \bar{Y}=3.36, \quad \quad P=0.124,\\ C_y&= {} 0.601, \quad C_p=2.678, \quad \rho _{pb}=0.766, \quad \beta _2(\phi )=6.612. \end{aligned}$$

The non-response rate in the population is considered to be 25 percent, taken as last 22 units of the population

$$\begin{aligned} N_2=22, \quad \quad \bar{Y}_2=3.27, \quad \quad C_{y(2)}=0.668. \end{aligned}$$

### Population II

[Source: Sukhatme and Sukhatme (1970), p. 256]

$$\begin{aligned} y&= {} \text {Area (in acres) under the wheat crop within the circles}.\\ \phi&= {} \text {A circle consisting of more than five villages}.\\ N&= {} 89, \quad \quad n=23, \quad \quad \bar{Y}=1102, \quad \quad P=0.124,\\ C_y&= {} 0.65, \quad C_p=2.678, \quad \rho _{pb}=0.624, \quad \beta _2(\phi )=6.612. \end{aligned}$$

The non-response units of the population are taken as last 22 units ($25\,\%$ of *N* )

$$\begin{aligned} N_2=22, \quad \quad \bar{Y}_2=1242.68, \quad \quad C_{y(2)}=0.516. \end{aligned}$$

The comparison is performed in terms of Percent Relative Efficiency (PRE)

$$\begin{aligned} \text {PRE}\left( \bar{y}^*_{(\bullet )}\right) =\dfrac{\text {MSE}\left( \bar{y}^*_{(\text {reg})}\right) }{\text {minMSE}\left( \bar{y}^*_{(\bullet )}\right) }\times 100 \end{aligned}$$

where $$\bar{y}^*_{(\bullet )}=\left( \bar{y}^*_\text {M1}, \; \bar{y}^*_\text {M2}, \; \bar{y}^*_\text {M3}, \; \bar{y}^*_\text {S1(1)}, \; \bar{y}^*_\text {S1(2)}, \; \bar{y}^*_\text {S2(R)}, \; \bar{y}^*_\text {S2(1)}, \; \bar{y}^*_\text {S2(2)}\right)$$.

**Table 1:** PRE of the estimators with respect to $\bar{y}^*_\text {reg}$ for different values of *k* for Pop I

Estimator	$\gamma$	$\delta$	$\eta$	$\psi$	*k*			
					2	3	4	5
$\bar{y}^*_{\text {M}2}$	–	–	$C_p$	$\beta _2(\varphi )$	100.94	101.39	101.85	102.30
	–	–	$\beta _2(\varphi )$	$C_p$	100.95	101.41	101.87	102.33
	–	–	1	$C_p$	100.94	101.39	101.85	102.30
	–	–	1	$\beta _2(\varphi )$	100.94	101.39	101.85	102.30
$\bar{y}^*_{\text {S}2(R)}$	–	–	1	0	100.94	101.39	101.85	102.30
$\bar{y}^*_{\text {S}2(1)}$	–	–	1	0	101.22	101.81	102.40	102.99
$\bar{y}^{*}_{\text {S}2(2)}$	–	–	1	0	118.34	114.94	113.55	112.93
$\bar{y}^*_{\text {M3}}$	1	1	*n*	$1-n/N$	114.65	112.36	111.49	111.18
	1	1	*N*	*P*	125.51	120.09	117.82	116.73
	1	1	*N*	$k_p$	125.21	119.88	117.65	116.58
$\bar{y}^*_{\text {M1}}$	–	–	1	0	126.32	120.65	118.27	117.11
$\bar{y}^*_{\text {S}1(1)}$	–	–	1	0	126.32	120.65	118.27	117.11
$\bar{y}^{*}_{\text {S}1(2)}$	–	–	1	0	*135.75*	*126.43*	*122.47*	*120.40*

From Tables 1 and 2, it is observed that the estimator $\bar{y}^*_{\text {M}2}$ with different values of $\eta$ and $\psi$ performs similar like $\bar{y}^*_{\text {S2}(R)}$. It should be noted that the estimator $\bar{y}^*_{\text {S2(2)}}$ with exponential function perform better than the estimators $\bar{y}^*_{\text {M}2}$ and $\bar{y}^*_{\text {S2(1)}}$ with ratio function. After careful analysis of performance of $\bar{y}^*_{\text {M3}}$, it is observed that different possible values of $\eta$ and $\psi$ increase the efficiency of the estimator. For $(\eta =1, \; \psi =0)$, the estimators $\bar{y}^*_{\text {M1}}$ and $\bar{y}^*_{\text {S1(1)}}$ perform similar as expected. The PRE of $\bar{y}^{*}_{\text {S}1(2)}$ is higher than those of $\bar{y}^*_{\text {M1}}$, $\bar{y}^*_{\text {M3}}$ and $\bar{y}^{*}_{\text {S}1(1)}$ which leads to the conclusion that exponential function may be a better choice than ratio. It can be seen that as the inverse sampling rate *k* increases, the PREs of the estimators $\left( \bar{y}^*_{\text {M}2}, \; \bar{y}^*_{\text {S}2(R)}, \; \bar{y}^*_{\text {S}2(1)}\right)$ also increase but the PREs of $\left( \bar{y}^*_{\text {M}1}, \; \bar{y}^*_{\text {M}3}, \; \bar{y}^*_{\text {S}2(2)}, \; \bar{y}^*_{\text {S}1(1)}, \; \bar{y}^*_{\text {S}1(2)}\right)$ decrease.

It has been shown in Singh and Solanki (2012) that the estimator $\bar{y}_{\text {SG}}$ with $(\eta =1, \; \psi =0)$ performs better than the estimator $\bar{y}_{\text {SS}}$ with complete information on *Y* . The same behavior is observed for $\left( \bar{y}^*_{\text {M1}}, \; \bar{y}^*_{\text {M3}}\right)$ in case of incomplete information on *Y* , in Table 1 and Table 2. Hence, from practical point of view, $\bar{y}^*_{\text {M1}}$ is preferable than $\left( \bar{y}^*_{\text {M2}}, \;\bar{y}^*_{\text {M3}}\right)$ because it is showing higher efficiency by using less auxiliary information as compared with others.

**Table 2:** PRE of the estimators with respect to $\bar{y}^*_\text {reg}$ for different values of *k* for Pop II

Estimator	$\gamma$	$\delta$	$\eta$	$\psi$	*k*			
					2	3	4	5
$\bar{y}^*_{\text {M}2}$	–	–	$C_p$	$\beta _2(\varphi )$	101.20	101.56	101.93	102.29
	–	–	$\beta _2(\varphi )$	$C_p$	101.21	101.58	101.95	102.32
	–	–	1	$C_p$	101.20	101.56	101.93	102.29
	–	–	1	$\beta _2(\varphi )$	101.20	101.56	101.93	102.29
$\bar{y}^*_{\text {S}2(R)}$	–	–	1	0	101.20	101.56	101.93	102.29
$\bar{y}^*_{\text {S}2(1)}$	–	–	1	0	101.56	102.03	102.51	102.98
$\bar{y}^{*}_{\text {S}2(2)}$	–	–	1	0	115.99	114.28	113.40	112.94
$\bar{y}^*_{\text {M3}}$	1	1	*n*	$1-n/N$	111.48	110.64	110.29	110.18
	1	1	*N*	*P*	118.59	116.52	115.45	114.88
	1	1	*N*	$k_p$	118.48	116.43	115.37	114.81
$\bar{y}^*_{\text {M1}}$	–	–	1	0	119.10	116.93	115.81	115.20
$\bar{y}^*_{\text {S}1(1)}$	–	–	1	0	119.10	116.93	115.81	115.20
$\bar{y}^{*}_{\text {S}1(2)}$	–	–	1	0	*128.32*	*123.85*	*121.40*	*119.92*

## Conclusions

In this paper, two new generalized classes of biased estimators for the population mean have been proposed when information on the auxiliary attribute is available, along with considering the problem of non-response on the study variable. Further, three modified classes of estimators motivated by Shabbir and Gupta (2007), Koyuncu (2012) and Singh and Solanki (2012) have also been considered in presence of non-response. Henceforth, linear regression estimator is considered as benchmark for comparing efficiency of the proposed classes. Our suggested classes $\bar{y}_{\text {S1}}$ and $\bar{y}_{\text {S2}}$ depend on the choice of function *g* and for this we consider ratio and exponential functions. Numerical results are reported in Tables 1 and 2 to show superiority of the suggested classes with the regression estimator. The main purpose of this paper is to highlight the non-response problem in the study variable when information of auxiliary attribute is available for estimating the unknown population mean.
